# Retinal gene therapy using epiretinal AAV-containing fibrin hydrogel implants

**DOI:** 10.1126/sciadv.adv7922

**Published:** 2025-09-05

**Authors:** Brittni A. Scruggs, Aubrey Berger, Travis Knudsen, Francesca N. Kopp, Matthew Hill, Emma Trncic, Kjersten Anderson, Raymond Iezzi, Alan D. Marmorstein

**Affiliations:** ^1^ Department of Ophthalmology, Mayo Clinic, Rochester, MN, USA.; ^2^ Department of Pediatrics, Mayo Clinic, Rochester, MN, USA.; ^3^ Graduate School of Biomedical Sciences, Mayo Clinic, Rochester, MN, USA.

## Abstract

Subretinal injection of adeno-associated virus (AAV) is generally more efficacious and less inflammatory than intravitreal injection for retinal gene therapy. However, adverse events (e.g., chorioretinal atrophy) have been reported in many patients receiving subretinal injection of Luxturna (voretigene neparvovec-rzyl) and experimental gene therapies. Subretinal injection confines transduction to the treated area. To address this, we manufactured high-concentration fibrin hydrogels encapsulating AAV2–green fluorescent protein (AAV2-*GFP*). Gels had homogeneous AAV distribution, desired mechanical properties, and retained infectivity. Epiretinal placement of fibrin–AAV2-*GFP* (*n* = 11) was compared to subretinal (*n* = 5) and intravitreal AAV2-*GFP* injection (*n* = 3). The subretinal group exhibited inconsistent retinal pigment epithelium (RPE) transduction restricted to the injection region with severe atrophy in two cases. The intravitreal group had weak transduction and inflammation. In contrast, epiretinal hydrogels degraded within days and led to broad transduction of RPE without atrophy or inflammation. This technology could advance gene therapy for retinal degenerations and other ocular or systemic disorders.

## Teaser

Fibrin hydrogels encapsulating adeno-associated virus transduce the retinal pigment epithelium when placed on the retina in pigs.

## INTRODUCTION

The Food and Drug Administration (FDA) approval of Luxturna (voretigene neparvovec-rzyl) for treatment of *RPE65*-related inherited retinal dystrophy (RPE65-IRD) in 2017 was the first regulatory approval of an in vivo gene therapy in the United States (*1*). Luxturna set the precedent for numerous investigational retinal gene therapies. It uses adeno-associated virus (AAV) as a delivery vector and is administered as a subretinal injection between the retinal photoreceptors and the retinal pigment epithelium (RPE), creating a temporary retinal detachment (“bleb”). Although the patient experience has been generally positive after Luxturna (*2*–*5*), there are several major limitations and challenges of administering any retinal gene therapy by subretinal injection.

Many inherited retinal diseases (IRDs) have abnormal retina-RPE adhesions that make subretinal delivery difficult, necessitating numerous blebs and higher than ideal pressure/flow in the subretinal space that likely damages RPE cells (*6*). Britten-Jones *et al.* found chorioretinal abnormalities to be the primary adverse event associated with subretinal injection across all human retinal gene therapies (*7*). Despite subretinal injections being performed by credentialed surgeons at select gene therapy centers, chorioretinal atrophy (CRA) is common (*5*, *8*–*12*) with reports indicating CRA occurs in 10.3 to 28.2% of eyes (*5*, *10*, *12*) in patients receiving Luxturna. CRA and other vision-threatening adverse events [e.g., macular holes (*5*) and inflammation (*13*–*15*)] are postulated to result from mechanical issues arising from detaching an unhealthy retina (*5*, *6*, *8*–*12*, *16*). We have shown that mechanical RPE debridement in pigs leads to rapid, progressive CRA (*17*), and we and others (*18*–*20*) have previously observed that subretinal injection of saline solution led to profound RPE changes in pigs, nonhuman primates, and rabbits, supporting the notion that subretinal injection is not the ideal route for retinal gene therapy. Despite the limitations associated with this technique, subretinal injection remains the preferred approach for gene therapy delivery for diseases affecting the RPE or photoreceptors given the superior access to the posterior pole of the eye compared to intravitreal or suprachoroidal injections, which are associated with an even higher incidence of intraocular inflammation (*21*, *22*). To address these issues, we sought to develop a means to deliver AAV to the outer retina without the need for subretinal injection.

Our approach involves adaptation of high-concentration (>30 mg/ml) fibrin hydrogels to deliver AAV vectored gene therapy; these hydrogels were originally developed for use in RPE transplantation (*23*–*26*). Fibrin forms a network of microfibrils that are the matrix of a blood clot. These microfibrils rapidly polymerize following cleavage of fibrinogen to fibrin by the enzyme thrombin. When performed in isolation, the microfibrils form a homogeneous hydrogel. At physiological concentrations (3 to 5 mg/ml), fibrin is difficult to handle and poorly suited to our intended purpose. However, at concentrations of >30 mg/ml, fibrin has sufficient rigidity to be handled and placed during surgery without damage to the hydrogel, and the hydrogels are sufficiently elastic to allow contact with the retina without damaging retinal tissues. Fibrin is a biocompatible substance with a safe track record of human clinical and ophthalmic use, and it is readily degradable in vivo due to the action of the enzyme plasmin. These properties, combined with the prior use of fibrin as a drug delivery vehicle (*27*, *28*), drove the current study in which we adapted these high-concentration fibrin scaffolds to deliver AAV vectored gene therapy to the RPE.

Herein, we detail the manufacturing of shaped high-concentration fibrin gels with AAV2 encapsulation, the in vitro characterization of these gels, and the transduction efficacy and safety outcomes associated with the surgical delivery of these gels to the epiretinal surface of domestic pigs compared to the conventional subretinal and intravitreal approaches.

## RESULTS

### CRA and transduction issues after subretinal AAV injection

**Fig. 1.**
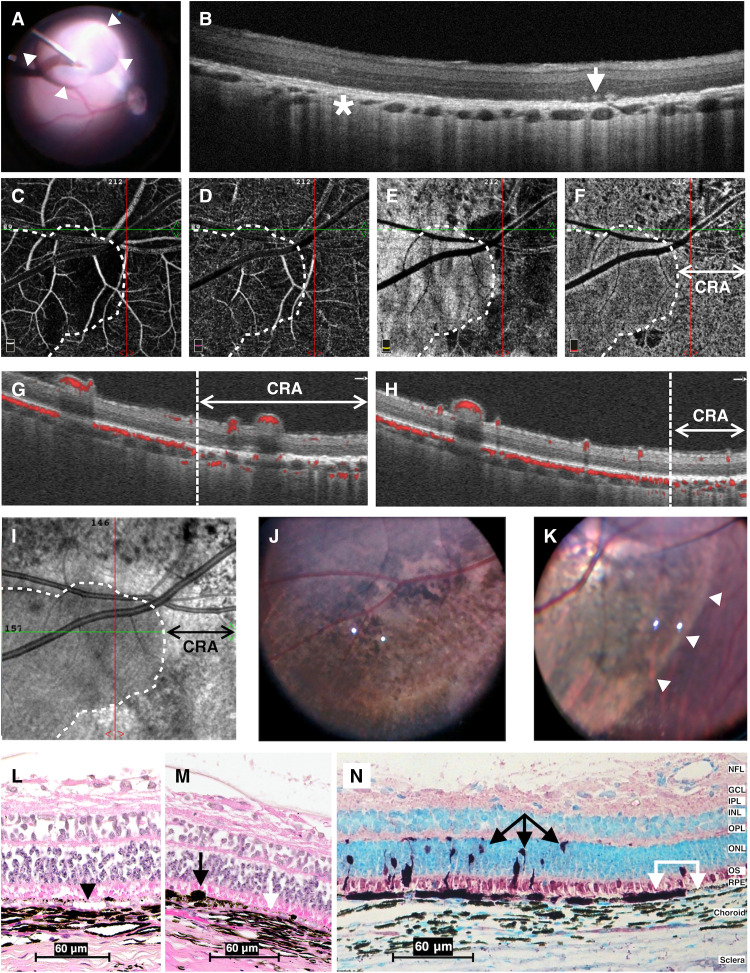
CRA and inconsistent RPE transduction 1 month after subretinal delivery of AAV2-GFP in pigs. (**A**) Intraoperative photograph shows cannula delivering AAV2 solution (bleb, arrowheads) into the subretinal space. (**B**) OCT through the area of subretinal injection demonstrates choriocapillaris loss (asterisk) and subretinal deposits with RPE loss (arrow). (**C** to **F**) OCT-A en face images show superficial (C), deep (D), outer retina (E), and choriocapillaris (F) vascular layers. Dashed lines outline the CRA extent (right of line) within the bleb. (**G** and **H**) OCT-A images of two different pigs demonstrate normal choriocapillaris flow outside the bleb and minimal to no choriocapillaris flow within CRA area. (**I**) Near-infrared confocal SLO imaging demonstrates increased reflectance correlating to atrophy and decreased reflectance correlating to deposits within the bleb (right of line). (**J** and **K**) Fundus photographs of two pigs with severe CRA within the bleb region [(K); arrowheads]. (**L** and **M**) H&E of retina through the area of CRA within the bleb. There is RPE vacuolation [(L); black arrowhead], hyper- and hypopigmentation [(M); white arrow], and occasional RPE hypertrophy [(M); black arrow]. (**N**) GFP signal using IHC staining reveals inconsistent RPE transduction by AAV2-*GFP* (white arrows) and transduction of photoreceptors (black arrows) within the bleb. Retinal layers are labeled as follows: NFL, nerve fiber layer; GCL, ganglion cell layer; IPL, inner plexiform layer; INL, inner nuclear layer; OPL, outer plexiform layer; ONL, outer nuclear layer; OS, outer segments of photoreceptors; and RPE. Scale bars, 60 μm.

Subretinal administration of AAV2–green fluorescent protein (AAV2-*GFP*) solution (Fig. 1A) led to progressive CRA with profound RPE changes (Fig. 1, B to M) in two of the five eyes treated. This occurred despite our subretinal blebs being performed as recommended for Luxturna administration with slow administration over several minutes and despite the AAV titer being twofold less than used in clinical practice. The extensive CRA was confined within the area of the subretinal bleb, seen as well-demarcated areas of hypopigmentation (i.e., atrophy) on fundus exam (Fig. 1, J and K) and by scattered overlying RPE clumping (Fig. 1B) observed using optical coherence tomography (OCT). OCT-angiography (OCT-A) (Fig. 1, C to H) demonstrated reduced blood flow in the choriocapillaris in regions of CRA in treated eyes. Increased reflectance correlating to atrophy and decreased reflectance correlating to deposits within the bleb were observed using scanning laser ophthalmoscopy (SLO) (Fig. 1I). Postmortem hematoxylin and eosin (H&E) staining confirmed CRA and identified RPE vacuolation, hypo- and hyperpigmentation, atrophy, and occasional hypertrophy with thinning of the choroid within these areas (Fig. 1, L and M).

We anticipated that subretinal injection of AAV2-*GFP* would result in high GFP expression in RPE cells, yet there was inconsistent RPE and photoreceptor transduction (Fig. 1N). In all subretinal pigs, immunohistochemical (IHC) staining for the GFP reporter showed photoreceptors and RPE cells that exhibited high expression of GFP immediately adjacent to cells with no transduction (Fig. 1N). As expected, transduction was confined to the bleb region (i.e., no peripheral retinal/RPE transduction) with the subretinal approach (table S1). These findings provided a model against which we could compare other approaches to gene therapy delivery to the outer retina.

### Production of large fibrin hydrogels using injection molding

The goal of this study was to produce fibrin hydrogels that can be used for gene therapy delivery. As we move toward clinical trials, the reproducibility of gel characteristics, especially shape, becomes important as this dictates the size of the dose delivered and influences the rate of scaffold degradation and its mechanical properties. We have consistently used an oval gel of 1.5 mm by 5.0 mm by 0.2 mm in size for our therapeutic application, but we found that pressing gels as done previously (*23*) resulted in too much variability in gel thickness. Thus, we needed to develop a method for commercial-scale production of fibrin hydrogels suitable for our application and with tight dimensional tolerances.

**Fig. 2.**
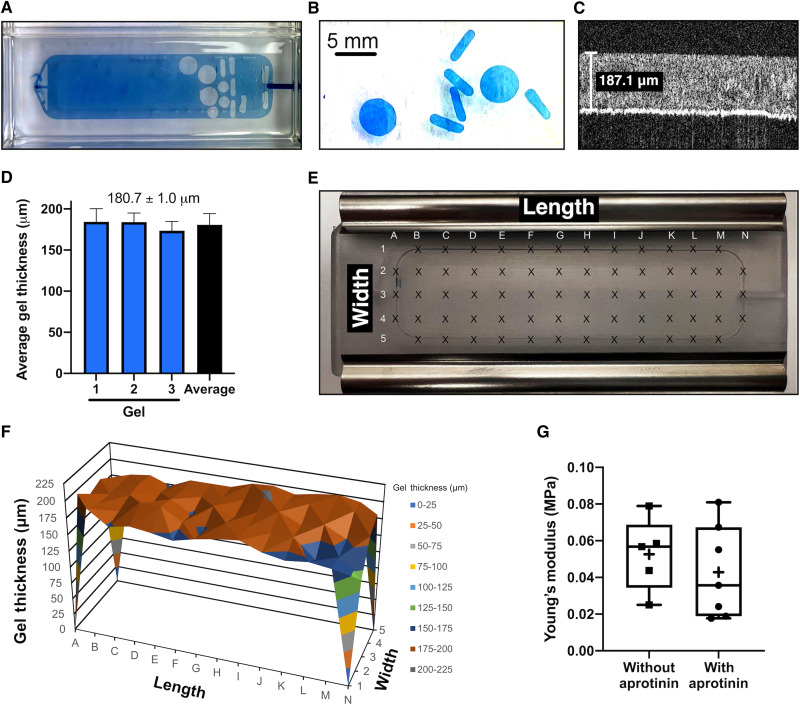
Fibrin hydrogel characterization by OCT and mechanical strength testing. (**A** and **B**) Addition of trypan blue slows fibrin polymerization facilitating injection molding of large–surface area gels with high-concentration fibrin (*26*). The mold (A) cavity is 15.25 mm by 58.42 mm by 0.2 mm in which a gel blank (blue) can be cast and from which a punch can produce smaller configured blanks or “punches” of various dimensions (B). (**C**) OCT demonstrates a gel with thickness of 187.1 μm. (**D** and **E**) Average ± SEM gel thickness of three different unpunched gels was determined using OCT imaging at the 66 designated points shown in (E). (**F**) Thickness of an unpunched gel determined by OCT at each of the tested 66 points; length and width correlate to the positions tested, shown in (E). (**G**) Gels made with or without aprotinin were tested for mechanical strength. Box plots represent 25th to 75th percentiles with vertical bars providing the range, horizontal bars represent median values, and crosses represent the mean value (*n* = 5 without aprotinin; *n* = 7 with aprotinin). Scale bar, 5 mm.

We have reported that the addition of trypan blue to the fibrin gelation mixture slows the initial polymerization of fibrin (*26*). We decided to use this property of trypan blue to manufacture fibrin hydrogels using injection molding (Fig. 2, A and B, and fig. S1). After going through a prototyping process, we arrived at a mold that resulted in fibrin hydrogel sheets that were uniformly of ~181 ± 1 μm (average ± SEM, *n* = 3) in thickness (Fig. 2, C to F) and could be used as blanks from which to punch different sized doses (Fig. 2, A and B). The fibrin gel mold is shown in fig. S1 and has two plates sealed with a silicon gasket held together by metal clips. The “top” plate is 28.57 mm by 78.36 mm, approximately the size of a microscope slide and has a cavity that is 15.25 mm by 58.42 mm by 0.20 mm in which the gel is formed. The top plate is machined from polycarbonate as we wanted a clear plate that would allow us to observe the filling of the mold. The inlet and outlet ports of the top plate were designed and placed to permit even filling and prevent formation of pockets or air bubbles during injection of the gelation mixture, to anchor the hydrogel in place, and to prevent the gel from being pulled from the bottom plate during disassembly of the mold after polymerization.

We chose to use a tapered dispensing tip with an inlet that is flush with the plate surface. This allows the plate to sit flat with the gel facing up, an advantage during casting as it allows the operator casting the gel to observe it filling. The “bottom” plate is machined from aluminum and has channels milled along both sides to hold the metal clips in place. The clips hold the entire mold assembly together. The gasket is a sheet of 0.794-mm (1/32″)–thick silicon. This thickness was found to be optimal; thicker gaskets allowed greater deformation under pressure, resulting in an unacceptable degree of variability in gel thickness. Earlier prototypes tested the use of a circumferential sealing gasket and a plastic bottom plate. However, this also resulted in unacceptable variability in thickness and sticking of the gel to the bottom plate.

The injection molding process uses a FibriJet 11:1 ratio applicator and blending connector. The 11:1 ratio permits maximal fibrinogen concentration, and the blending connector results in uniform mixing of the thrombin and fibrinogen solutions as they are injected into the mold (fig. S1). A 4-ml Tisseel kit, prepared as outlined in the methods, has sufficient volume to mold up to three 15.25 mm–by–58.42 mm–by–0.2 mm gel blanks.

### Mechanical characteristics of injection-molded fibrin hydrogels

The ability to use a punch to generate smaller-shaped pieces of the hydrogel from 15.25 mm–by–58.42 mm–by–0.2 mm gel blanks indicated a mechanical stiffness sufficient to permit the use of punching to subdivide the gel into uniform pieces of smaller size (Fig. 2, A and B). OCT analysis indicated that the gel has sufficient elasticity to rebound at the edges to its original thickness as the average of a punch does not differ from the average of a blank. We next tested the hydrogel elasticity/compression characteristics. Mechanical strength testing of fibrin gels was similar without and with aprotinin with the Young’s modulus calculated as 0.053 ± 0.01 and 0.042 ± 0.01 MPa, respectively (Fig. 2G; *P* = 0.49).

### Dimensional and structural characterization of shaped fibrin hydrogels

To determine the uniformity and characteristics of the hydrogels, we examined several physical properties. Using OCT, we measured gel thickness and homogeneity. A representative OCT image is shown in Fig. 2C, demonstrating an average thickness of 180.7 ± 1.0 μm (Fig. 2D) across the 198 regions measured in three gels (Fig. 2F) with measurements made at 66 different regions of each gel blank (Fig. 2E). There was no significant variability in gel homogeneity by OCT across a single blank. Although occasional air bubbles were observed, they were infrequent and <1 mm in diameter.

**Fig. 3.**
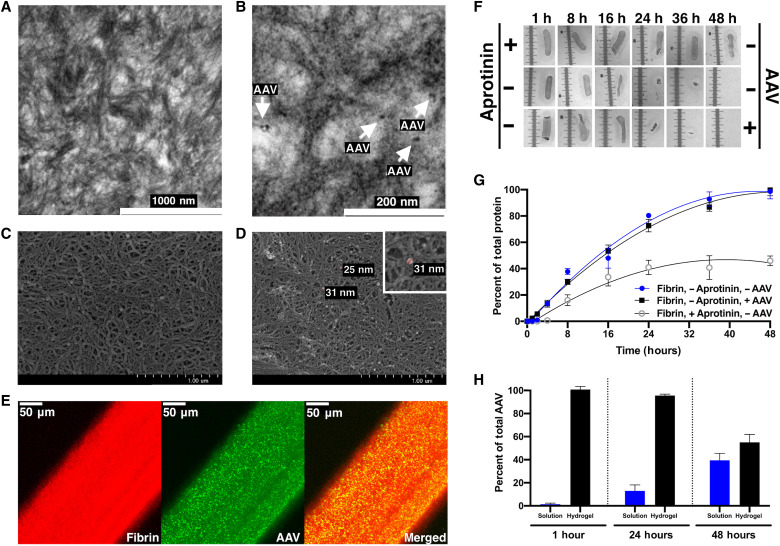
Characterization of fibrin-encapsulated AAV gels. TEM (**A** and **B**) and SEM (**C** and **D**) show dense arrays of fibrin microfilaments without AAV (A and C) and with AAV particles (B and D), which measure 25 to 31 nm. (**E**) IF staining demonstrates the homogeneity of fibrin (red) and AAV particles (green) in a gel containing 2 × 10^9^ AAV vg. (**F**) Serial photographs of fibrin gels over 48-hour period demonstrating plasmin-mediated degradation with addition of AAV or addition of aprotinin. h, hours. (**G**) Protein assay was used to measure plasmin-mediated degradation of fibrin either with AAV (black squares) or without AAV (blue circles). AAV had no effect on degradation. Addition of aprotinin delays fibrin degradation (white circles). (**H**) ELISA was used to determine the percentage of AAV released from gels or retained in gels at 1, 24, and 48 hours at 37°C. Data are presented as means ± SEM (*n* = 3 experiments). Scale bars, 50 μm.

Transmission electron microscopy (TEM) (Fig. 3, A and B) was performed to visualize the structure of fibrin in cross sections of the gels with and without AAV. Gels were found to have a fibrillar structure, similar to that which we have reported previously (*26*), with fibrils randomly oriented but generally uniform in diameter, length, density, and cross-linking density. Scanning electron microscopy (SEM) (Fig. 3, C and D) indicated that the hydrogel surface was similar to that which we observed previously (*23*, *24*, *26*). The surface architecture appeared to be composed of fibrils aligned parallel to the top surface plane with crater-like voids appearing fairly heterogeneously across the surface. In gels with AAV, some AAV particles could be observed along the surface fibers and within the craters (Fig. 3D).

### Even distribution and high density of AAV particles throughout the fibrin gel

As expected, AAV did not affect the gel structure or fibrin density, but individual particles of ~25 to 31 nm were consistently noted in both TEM (Fig. 3B, black particles) and SEM (Fig. 3D, white particles) with a homogeneous distribution; these particles are consistent with the size of AAV particles. In Fig. 3, TEM and SEM images are representative of three different fibrin gels, and the results suggest AAV particles evenly distribute throughout the gel with AAV particles residing within the spaces between fibrils. We further confirmed the presence, even distribution, and density of AAV particles throughout our gels by performing immunofluorescence (IF) staining for AAV capsids in fibrin-AAV gels (Fig. 3E).

### Modifying the gel enhances the rate of degradation

Fibrin gels were produced using the FDA-approved tissue glue, Tisseel. The components of the Tisseel kit include lyophilized fibrinogen and thrombin, allowing customization of the solution used to resuspend the lyophilized components. The buffer included in a Tisseel kit for resuspension of fibrinogen contains aprotinin, an antifibrinolytic protease inhibitor that slows the degradation of fibrin gels (Fig. 3, F and G). We replaced the solution used to resuspend fibrinogen with 0.01 M sodium citrate (pH 6.0); this resulted in a significant decrease in the time required for in vitro degradation by plasmin of the modified gel in comparison to gels prepared using the included resuspension buffer (*P* < 0.0001; Fig. 3, F and G). Gels containing aprotinin degraded to only ~50%, even after 48 hours, whereas gels cast with sodium citrate and no aprotinin were 100% degraded within 36 to 48 hours with or without AAV incorporated (Fig. 3F).

### AAV retains infectivity after release from fibrin gels

**Fig. 4.**
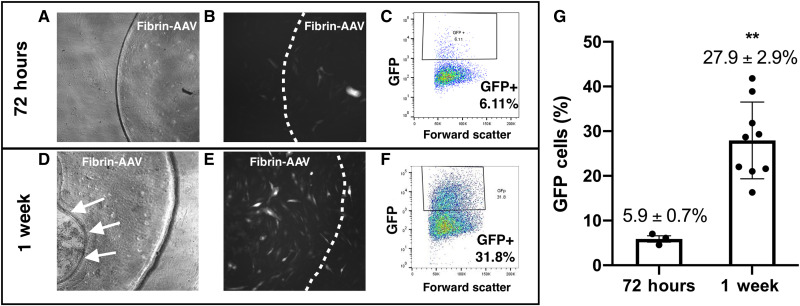
Retained infectivity of fibrin-encapsulated AAV2-*GFP*. A dose (7 × 10^9^) of AAV2-*GFP* incorporated into a 3-mm-diameter fibrin gel was incubated with confluent ARPE-19 cells and evaluated at 72 hours (**A** to **C**) and 1 week (**D** to **F**) by flow cytometry (C and F), bright-field microscopy (A and D), and fluorescence microscopy (B and E). There was increased transduction (fluorescent cells) at 1 week (E) compared to 72 hours (B). The fibrin gel is outlined by dashed lines. Arrow in (D) demonstrates partial degradation of the gel by week 1. (**G**) Flow cytometry demonstrates a significant increase in GFP expression between 72 hours and 1 week. Data are presented as means ± SEM. Representative flow cytometry data for a representative run are included in (C) and (F). **P < 0.01.

An enzyme-linked immunosorbent assay (ELISA) was used to follow diffusion/retention of AAV from the gel at 1, 24, and 48 hours at 37°C. We found that AAV slowly diffuses from fibrin gels (*n* = 7 gels) over a period of days with ~55% remaining in the gel at 48 hours (Fig. 3H); this confirmed that the virus does not immediately release from the gel when placed in solution. Longer time points (72 and 96 hours and 1 week) were attempted. However, ELISA results were unreliable at these later time points given that the virus was not stable long-term in solution. When applied in vivo, we expect AAV to be released primarily through degradation of the hydrogel. When fibrin-encapsulated AAV gels were placed in a 96-well plate of near-confluent ARPE-19 cells (Fig. 4, A to F), 5.9 ± 0.7% of cells were transduced by 72 hours with a significant transduction increase to 27.9 ± 2.9% at 1 week (Fig. 4G, *P* < 0.01), confirming retained RPE infectivity of fibrin-encapsulated AAV. Figure 4D shows the gel starting to degrade on top of the cells with well-defined regions of completely degraded hydrogel (arrows). Figure 4 (B and E) highlights the stronger transduction of the cells immediately under the implant compared to the surrounding area.

### Intraoperative procedure for fibrin-AAV epiretinal placement

**Fig. 5.**
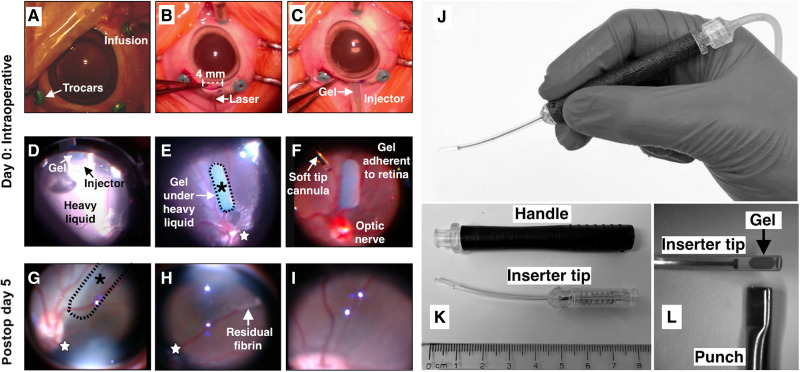
Surgical placement of fibrin-encapsulated AAV on the epiretinal surface. (**A**) Lens-sparing 25-gauge vitrectomy is performed. (**B**) A 4-mm scleral incision is made with blade to the choroid, which is then cauterized using a laser before full incision with keratome. (**C**) The inserter loaded with an oval AAV-fibrin implant (blue) is inserted. (**D**) The inserter tip is placed under PFO, and the implant is deployed. (**E**) Autologous serum is injected around the implant to glue it in place under PFO. (**F**) A soft tip cannula is used to exchange the PFO for air and then SF6 gas. The implant remains in place. (**G**) On day 5, dilated fundus examination confirms degradation of the hydrogel with no implant visualized at the original site (dashed oval and asterisk). (**H**) Minimal residual fibrin is noted on the retina surface on day 5, consistent with degradation of the implant. The small star provides the orientation across (E), (G), and (H). (**I**) One month postoperatively, the view is clear with no inflammation, no cataract, and no evidence of choroidal or retinal abnormalities, including atrophy and vasculitis. (**J**) Inserter used to place implant consists of two parts; (**K**) a handle and separate inserter tip. (**L**) Punched oval implant is visible in the inserter tip and ready for placement.

We developed a surgical procedure to place and adhere the fibrin gel to the epiretinal surface. Figure 5 (A to F) shows the main surgical steps involved in epiretinal placement of a fibrin-AAV hydrogel (movie S1) with follow-up imaging shown for postoperative day 5 (Fig. 5, G to I).

A three-port core vitrectomy with triamcinolone-assisted separation of the posterior hyaloid was performed with partial peripheral shave. Figure 5 (J to L) shows a custom injector we developed to deploy the gel into the eye via pneumatic foot pedal assist or manual injection; the gel is visible and ready for insertion in Fig. 5 (C and L). We found that deploying the gel under perfluoro-*n*-octane (PFO) (Fig. 5D) and adding autologous serum around the gel (Fig. 5E) leads to consistent adherence of the gel to the epiretinal surface (Fig. 5F). Across all 11 experimental epiretinal pigs, we had no issues with inserting gels or having the gels remain stuck to the area centralis even after PFO-to-air exchange and then air-to-gas exchange. We performed all epiretinal surgeries the same, each with autologous serum, to ensure consistency during protocol development.

### Epiretinal-placed fibrin degrades quickly without fibrin hydrogel-related complications

Color fundus photos, OCT, and OCT-A were performed at 5 to 7 days, 2 weeks, and 1 month following surgery for all pigs. By days 5 to 7, the gas bubble had dissipated in all 11 epiretinal pigs, and the fibrin gel had degraded (Fig. 5G). In two pigs, there was very minimal fibrin residue at this time point in the periphery (Fig. 5H), but this was completely resolved by week 2. Overall, the pigs tolerated the surgery well with no CRA and no severe inflammation (Fig. 5I). Three of the 11 pigs exhibited complications of surgery unrelated to the fibrin gel. One developed a suture abscess at week 2 at the site of the sclerotomy closure despite an unremarkable surgery, and postoperative course required 2 weeks of systemic and topical antibiotics. The pig never had distress, and the retina appeared healthy with no signs of clinical inflammation. A second pig had a postvitrectomy cataract develop during the 1-month study period; this limited postoperative photos and OCT analysis. A third pig was noted to have a localized retinal detachment in the periphery at the time of euthanasia. Given that there is no pars plana in the pig, peripheral retinal detachments are much more common than in the human patient, and this is the reason why we did not perform a thorough vitrectomy peripherally. Of these three pigs that had surgical complications, all had strong and diffuse GFP expression in the RPE with no retinal inflammation noted during clinical or postmortem examination. We attribute the minor complications noted to a lack of hygiene, the vitrectomy procedure, and pig-specific anatomy (e.g., lack of pars plana), not the fibrin. Pig outcomes and clinical findings of all study pigs are listed in table S1.

### Epiretinal fibrin-AAV transduces the RPE

**Fig. 6.**
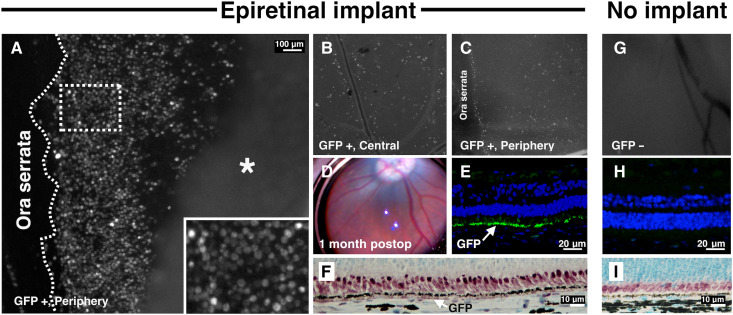
Strong RPE transduction detected 1 month after epiretinal placement of fibrin-encapsulated AAV hydrogels in 11 pigs. (**A**) Postmortem analysis of GFP expression shows strong transduction throughout the RPE (inset) when the retina (asterisk) is reflected 1 month after epiretinal gel placement. (**B** and **C**) Scattered cells exhibiting GFP expression within the neurosensory retina are observed throughout the posterior pole (B) and the periphery (C). (**D**) Normal fundus appearance at 1 month indicating no clinical adverse effects. (**E** and **F**) Histological analysis of the retina reveals GFP expression using IF staining (E) and IHC staining for GFP (F), consistent with RPE transduction by AAV2. (**G** to **I**) The fellow (control) eye has no GFP signal by postmortem analysis of GFP expression (G), IF staining (H), or IHC staining (I). Nuclei are stained with DAPI in (E) and (H) and methyl green in (F) and (I). Scale bars, 100 μm for (A), 20 μm for (E) and (H), and 10 μm for (F) and (I).

**Fig. 7.**
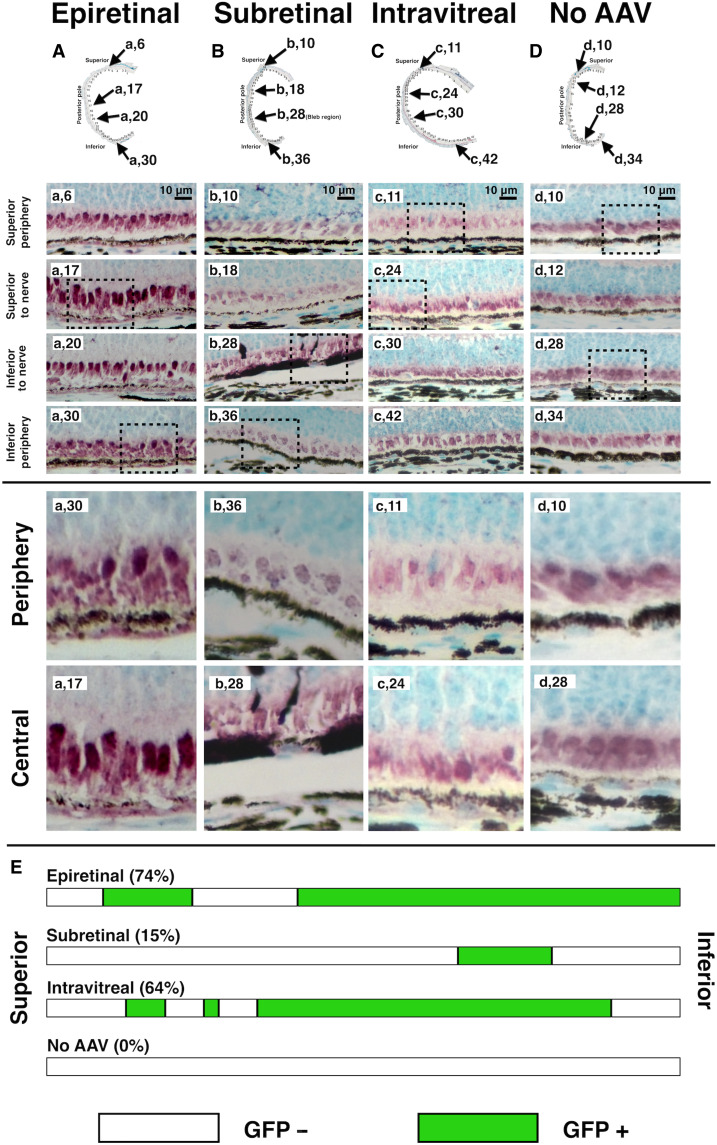
Extent of GFP expression in eyes following epiretinal, subretinal, and intravitreal delivery of AAV2-*GFP*. Histologic sections of paraffin-embedded eyes were cut along the inferior/superior axis and stained using IHC staining for GFP expression (**A** to **D**). Sections were photographed stepwise using a 10x objective from the superior ora serrata (section 1) to inferior ora serrata and assembled into a combined image covering the entire section. GFP staining in each section was visually evaluated, and representative sections are shown for epiretinal [(A); sections 6, 17, 20, and 30], subretinal [(B); sections 10, 18, 28, and 36], intravitreal [(C); sections 11, 24, 30, and 42], and fellow eye [(D); sections 10, 12, 28, and 34]. For each group, sections are designated by lowercase letter and retinal location (e.g., a,6 for epiretinal group A, section 6), and two dashed areas were magnified of the peripheral and central retina. (A) For the epiretinal group, consistent moderate GFP staining was observed in RPE cells in most sections from superior ora serrata to inferior ora serrata. (B) In the subretinal injection eyes, staining was observed in RPE and photoreceptors. GFP-expressing cells typically expressed strong staining but were often immediately adjacent to cells that did not express GFP. Staining was confined to sections b,27 to b,32, corresponding to the bleb region. (C) In the intravitreal group, GFP expressed was visualized at very modest levels in RPE cells compared to epiretinal or subretinal groups. (D) There was no GFP staining in any section of the fellow (control) eyes. (**E**) For each group, the retinal images/sections were individually graded from superior ora serrata (left) to inferior ora serrata (right) on a binary scale: GFP negative (−, white) or GFP positive (+, green). Total GFP positivity was calculated as a percentage of GFP-positive sections. Scale bars, 10 μm.

**Fig. 8.**
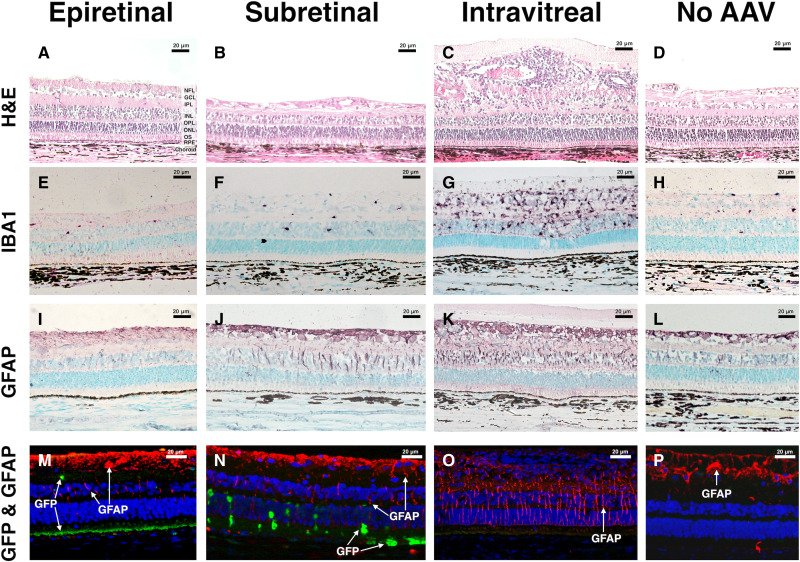
Histologic assessment of transduction, inflammation, and gliosis across three routes of AAV administration. AAV2-*GFP* was administered by the epiretinal-placed fibrin-encapsulated AAV gel (**A**, **E**, **I**, and **M**), subretinal injection (**B**, **F**, **J**, and **N**), and intravitreal injection (**C**, **G**, **K**, and **O**), or left untreated (**D**, **H**, **L**, and **P**). One-month postinjection eyes were processed for histology. Staining with H&E in epiretinal eyes (A) and those not receiving AAV (D) was unremarkable. RPE changes and CRA was noted in some subretinal eyes (B). RPE exhibits vacuolation and loss of pigment. Intravitreal eyes (C) exhibited notable inflammation with clear cellular infiltrates surrounding inner retinal blood vessels. IBA1 staining (E to H) did not differ in epiretinal and subretinal eyes from no AAV controls (H). However, the intravitreal injections led to a substantial increase in macrophage levels throughout the retina (G). Müller cell gliosis assessed by the presence of GFAP staining (I to P) in Müller cell projections in the INL and ONL was similar in epiretinal (I and M) and control eyes (L and P). The subretinal group had an apparent increase in Müller cell gliosis (J and N), which was prominent in the region of the bleb where cells were GFP positive (N). Intravitreal eyes exhibited notable Müller cell gliosis (K and O) throughout the retina even in regions with little or no GFP expression (O). Nuclei in (M) to (P) were stained with DAPI (blue). Nuclei in (E) to (L) were stained with methyl green. Retinal layers are labeled in (A): NFL, nerve fiber layer; GCL, ganglion cell layer; IPL, inner plexiform layer; INL, inner nuclear layer; OPL, outer plexiform layer; ONL, outer nuclear layer; OS, outer segments of photoreceptors; and RPE. Scale bars, 20 μm.

We next qualitatively compared GFP expression in eyes receiving epiretinal AAV-*GFP*. When the retina was peeled back on flat-mounted sections in the epiretinal group, there was diffuse expression of GFP fluorescence in hexagonal cells, consistent with RPE (Fig. 6A). The epiretinal fibrin gels consistently transduced the RPE both centrally and in the periphery (Fig. 7, A and E), demonstrating that concentrating AAV therapy on the retinal surface under gas allows the vector to pass through the inner limiting membrane (ILM) and entire retina to selectively transduce the RPE—not only around the initial gel placement. None of the epiretinal pigs had evidence of clinical inflammation (Fig. 6D, representative fundus imaging at month 1). Furthermore, all epiretinal pigs had RPE GFP transduction confirmed by some combination of GFP fluorescence observed in whole-mounted eyes (Fig. 6, A to C), IF staining (Fig. 6E), and/or IHC staining (Figs. 6F, 7A, and 8M). The fellow eyes of the epiretinal pigs had no GFP expression by any of these methods (Figs. 6, G to I, 7D, and 8P).

### GFP expression in epiretinal fibrin-AAV, subretinal injection, and intravitreal injection groups

We compared the distribution of GFP-positive cells in eyes receiving epiretinal fibrin-AAV gels (*n* = 11) with those receiving subretinal injection (*n* = 5) or intravitreal injection (*n* = 3). In the epiretinal group, RPE cells expressing GFP were observed in most of the eye with expression covering an area extending from the inferior to the superior ora serrata (Fig. 7, A and E). Most RPE cells exhibited similar, although modest, levels of GFP staining. Occasional cells in the inner retina were observed to express GFP as well (Figs. 6, B and C, and 8M), but the frequency was low. In contrast, GFP expression in the subretinal group was noted to be confined to the region of the bleb (Fig. 7, B and E). GFP expression in this region was irregular with small stretches or even single very strongly expressing cells immediately adjacent to cells that exhibited no GFP staining (Figs. 1N, 7B, and 8N). In many areas within the bleb, photoreceptors were also expressing GFP (Figs. 7B and 8N). Eyes receiving intravitreal injection exhibited faint, inconsistent, and very limited GFP expression (Figs. 7C and 8O), which was not confined to any particular region.

### Fibrin-encapsulated AAV gene therapy delivery does not incite retinal inflammation

There was no clinical evidence of inflammation (i.e., no vasculitis, snowballs, hypopyon, etc.) in the epiretinal or subretinal groups for all pigs that could be examined postoperatively (i.e., no cataract), In the intravitreal group, two eyes had severe clinical inflammation and one had mild retinal vascular sheathing, consistent with mild vasculitis (table S1). The two pigs that received intravitreal injection and exhibited severe vitritis at week 1 also had cataract formation by month 1. Neither of the pigs with vitritis was noted to be in pain; they were started on frequent topical steroid drops. One of the pigs required daily systemic steroids (during postoperative week 2) given the severity of the inflammation. These topical and systemic steroid protocols resolved the injection (redness), anterior chamber cell, and vitritis, but month 1 images were limited by cataract. For the intravitreal pig that had the most inflammation, an anterior chamber tap was performed at week 1, before systemic steroids, to rule out infection. This sample tested negative by Gram staining and bacterial and fungal culture.

Inspection of H&E-stained sections from epiretinal and subretinal groups indicated no obvious inflammation. However, eyes from pigs in the intravitreal injection group exhibited regional thickening of the inner retina with a clear cellular infiltrate and an epiretinal deposit indicative of a strong immune response (Fig. 8 C, G, K, and O). Staining of ionized calcium-binding adaptor molecule 1 (IBA1) (Fig. 8, E to H) expression demonstrated no notable difference in epiretinal or subretinal eyes versus untreated fellow eyes. In contrast, eyes in the intravitreal injection group exhibited noticeably increased IBA1-positive cells, likely microglia. Eyes from the epiretinal group exhibited glial fibrillary acidic protein (GFAP) staining (Fig. 8, I and M) that was similar to that of the uninjected control eyes (Fig. 8, L and P). Treated eyes from the subretinal and intravitreal groups both exhibited prominent GFAP staining in Müller cell bodies extending from the inner retina toward the outer retina (Fig. 8, J and K). This was confined to the bleb region in subretinal eyes but was also observed in the peripheral retina in eyes from the intravitreal injection group. This suggests an increased number of activated Müller cells in subretinal (Fig. 8, J and N) and intravitreal (Fig. 8, K and O) versus epiretinal treated eyes (Fig. 8, I and M).

### No anterior segment or systemic detection of AAV postoperatively

The cornea, iris, lens, vitreous, optic nerve, and liver were collected following euthanasia and tested by quantitative polymerase chain reaction (qPCR) for AAV genomic DNA. Table S2 shows qPCR results for both eyes and liver when the sample was available for testing. Results were indexed against the limit of detection determined for the assay through spike-ins (fig. S2 and table S2). There was no AAV detected in any liver sample or any fellow eye sample. For experimental eyes, there was no AAV detected in iris, cornea, lens, or optic nerve samples. The vitreous samples of the epiretinal pigs had AAV detected in three experimental eyes, two of which had high levels detected. The subretinal and intravitreal qPCR data were limited due to the use of eyes for histology purposes; however, two liver samples from pigs receiving subretinal injection and three liver samples and two optic nerve samples from pigs receiving intravitreal injection were tested with no AAV detected.

### Safe use of fibrin hydrogels in human macular hole surgery

A similar fibrin hydrogel (5-mm diameter round) was used to cover a chronic, recurrent macular hole in the eye of a human patient (fig. S3). After 5 days, the gel was almost degraded completely, and by 12 days, the gel had completely degraded. The patient had a significant improvement in retinal anatomy, including at the 26-month follow-up. No inflammation or damage was noted. Similarly, we noted no retinal injury in any of the pigs we treated with this epiretinal approach.

## DISCUSSION

Previous studies from our group (*6*, *17*, *18*) and others (*8*, *10*, *11*, *16*, *29*) have demonstrated the limitations and risks of subretinal gene therapy. A critical finding is that transduction of target cells is limited to the area of the bleb (*30*). This prevents rescue in as much as 80% of the retina, a significant problem for diseases such as retinitis pigmentosa that progress from the peripheral to central retina. Furthermore, adverse effects due to subretinal injection put the patient’s vision and success of the therapy at risk. For example, perifoveal CRA following subretinal gene therapy injection is now recognized as a common complication in human surgeries for Luxturna administration (*8*, *10*) and has been reported in multiple human studies (*5*, *8*, *10*, *11*, *29*) and various animal models (see Fig. 1). To create a subretinal bleb, the abnormally adherent retina of patients with outer retinal degenerations must be detached requiring faster injection speeds or higher injection pressures, both of which have been associated with significant loss or damage to RPE and photoreceptors in pig (*18*) and monkey eyes (*19*). Furthermore, elevated injection pressures may increase flow-related complications, such as overstretching or thinning of the retina, secondary macular hole, or excessive egress of the vector solution (*31*). Avoidance of these adverse events is paramount to the success of gene therapy trials.

In the rapidly advancing field of gene therapy for ocular diseases, a safer and more effective alternate to subretinal injection is urgently needed to avoid the dangers of concentrating viral solution under a surgically detached retina. One approach that has been proposed is injecting the viral solution beneath the ILM (sub-ILM). However, sub-ILM injection is a technically challenging approach that creates a localized bleb between the ILM and the nerve fiber layer (*32*). The result is that the gene therapy is confined to the bleb, much like what occurs with subretinal injection. Intravitreal injection has had generally poor efficacy outcomes in human clinical trials and is associated with an increased frequency and severity of inflammation (*33*–*37*); our results corroborate this with weak RPE transduction and increased inflammation in the intravitreally treated animals. Suprachoroidal injection has also been associated with significant inflammation in animal models (*22*, *38*), and no results from a human gene therapy clinical trial data have been reported to date.

To address these issues, we developed a means to deliver AAV to the outer retina without the need for subretinal injection using AAV encapsulated in a high-concentration fibrin hydrogel. Like subretinal injection, this method is surgical, distinguishing it from intravitreal and suprachoroidal injections, which can be carried out in an exam room setting. Unlike subretinal injection, there is no need to detach the retina. From the standpoint of inflammation, epiretinal fibrin-AAV gels appear to induce the least amount of inflammation when compared to equivalent doses of AAV2-*GFP* delivered subretinally or intravitreally (Fig. 8).

The manufacture of fibrin hydrogels at high concentrations is difficult due to the very rapid (1 to 2 s) polymerization of fibrin and the viscosity of the fibrinogen containing solution. Our approach to this takes advantage of our recent finding that trypan blue or other select azo dyes added to the gelation mix extended the initial gelation time for high-concentration fibrin gels (*26*). This addition permits the production of large high-concentration fibrin hydrogels by injection molding. The gels produced cause no damage or disruption to the retinal tissue (*24*), unlike subretinal or sub-ILM approaches, because the fibrin gel has a Young’s, or elastic, modulus similar to that of the retina and was designed this way intentionally to minimize the potential for mechanical damage on placement due to contact with the retina. When adhered to the epiretinal surface, the AAV is released through a combination of diffusion and fibrin degradation (Figs. 3 and 4) with retention of infectivity when tested in vitro (Fig. 4) and in vivo (Figs. 6 to 8).

Although others have studied the use of fibrin hydrogels to deliver gene therapy, prior studies have either mixed the fibrin with other nondegradable scaffold components or used the gel in a form that is practical only for in vitro use. For example, Lee *et al.* studied fibrin for cartilage tissue engineering and showed that incorporating AAV2-*GFP* into fibrin tissue glue led to varying levels of transduction of human embryonic kidney (HEK) 293 cells, with higher pore sizes leading to more vectors released (*28*). In that study, however, degradation of the fibrin gels did not occur, likely due to the presence of aprotinin, which we have removed from our gelation mix. In 2022, Graceffa demonstrated fibrin-mediated delivery of plasmid DNA in cultured dermal fibroblasts (*27*). Neither of those studies demonstrated gene transfer in animals, tissue, or terminally differentiated cells. The later study did not examine a viral-based gene therapy and had very low transfection efficiency. Furthermore, the cells were passaged onto a gel that was formed in a tissue culture plate. That was presumably because fibrin at a low concentration (3 mg/ml) has such a soft consistency that it is difficult to handle (*27*).

In contrast, our high-concentration gels are (i) portable, (ii) can be cast and punched or cut to any desired shape, (iii) are easy to handle, (iv) readily degrade, and (v) can be used with AAV to transduce terminally differentiated cells in an animal with high efficiency. To further advance this technology for ophthalmic and nonophthalmic applications, we have used molded blanks from which we can produce smaller gels of precise dimensions that correspond to an individual dose for a gene therapy application (Fig. 2). We have shown that oval-shaped fibrin gel punches placed on the epiretinal surface completely and safely degrade within days (Fig. 5 and fig. S3). This is likely due to the presence of endogenous plasmin in the eye. Although this differs from our prior studies demonstrating fibrin degradation over weeks when placed in the subretinal space for RPE transplantation (*24*), those gels were degraded in a similar time frame if the endogenous RPE had been previously debrided.

The surgical placement of epiretinal hydrogels is a relatively simple procedure. The only step that would require additional training for a fellowship trained vitreoretinal surgeon would be the insertion of the gel through the sclerotomy and deploying the gel under the PFO. All other procedures used are typical of routine vitrectomy. A critical aspect of our surgical approach is placement of the gel on the retinal surface under a gas tamponade. This allows the virus to slowly release from the hydrogel and to concentrate in the crescentic space between the gas bubble and the retina, presumably driving diffusion of the AAV particles into the retina. This has the effect of preventing free dispersal of vector throughout the vitreous cavity as might occur with an intravitreal injection and abolishes the need to detach an already unhealthy retina. Our approach facilitates the gradual dissolution of the gel over days while also concentrating the virus near the retina, potentially decreasing the viral titer required for efficacy. A result of this is that we observed little or no inflammation in the 11 eyes studied and generally did not detect viral genomes (vg) outside of the retina. This suggests that this technology is both safe and, on the basis of the broad and uniform expression of GFP in RPE cells, effective. The absence of photoreceptor transduction as well as the apparent absence of posttreatment inflammation in these pigs may be, in part, due to different concentrations of vector particles/genomes at the level of the outer retina compared to those obtained with subretinal delivery.

The pigs receiving subretinal versus epiretinal AAV had many differences that are clinically relevant. In eyes receiving subretinal AAV at comparable titers, GFP expression was confined to the bleb. Within the bleb, transduction of both RPE and photoreceptors was observed with high variability in GFP expression. In some cases, cells expressing very high levels of GFP were immediately adjacent to areas of apparently nontransduced cells. This occurred despite the moderate titer (2.5 × 10^9^) used and poses a concern that transgene overexpression could possibly contribute to retinal toxicity in subretinal blebs, especially at higher titers (e.g., 1.5 × 10^11^, the dose used for Luxturna). The epiretinal group, in contrast, exhibited a uniform, acceptable level of RPE transduction throughout the RPE with little or no photoreceptor expression observed (Figs. 6 to 8). In contrast to the subretinal eyes, epiretinal eyes exhibited widespread GFP expression from posterior pole to ora serrata (Fig. 8). GFP expression was not confined to the region of the retina on which the hydrogel was adhered. This supports the hypothesis that virus under the gas bubble traversed across the retina in all quadrants. Increased size of the transduction area—a welcomed result of this study—may protect at-risk peripheral retina that conventional methods currently leave untreated. Last, eyes receiving subretinal AAV had increased GFAP positivity throughout the bleb region with GFAP detected from the inner retina to the external limiting membrane; the same pattern of localization was not seen in the epiretinal or untreated groups. This was an expected finding as it is known that retinal detachment leads to glial activation and other potential complications (*39*).

In addition to subretinal injection, gene therapy trials have tested injection into the vitreous and the suprachoroidal space (between the choroid and sclera). Although there are active clinical trials (NCT04514653 and NCT04567550) using suprachoroidal injection, no outcomes have yet been reported. However, recent animal studies suggest that there is increased inflammation after suprachoroidal AAV injection, likely due to high uveoscleral outflow (*22*, *38*, *40*) and thus leakage of vector into the general circulation. In a study of AAV vector delivery method versus inflammatory response, Wiley *et al.* (*22*) observed that the degree and frequency of inflammation correlated with route of delivery. Intravitreal and suprachoroidal injections had the greatest local inflammation.

Although intravitreal injections are routinely performed on an outpatient basis for treatment of common disease states (e.g., age-related macular degeneration) (*41*), intraocular inflammation, which is not common in patients receiving intravitreal injection of other therapeutics, has been the primary adverse effect of intravitreally injected AAV therapy in preclinical and clinical trials (*7*, *35*). Across four independent trials of *ND4-*related Leber hereditary optic neuropathy, uveitis was reported in 42 of 84 eyes receiving intravitreal AAV (*7*, *37*). Four of the nine treated eyes had inflammation after intravitreal AAV gene therapy for X-linked retinoschisis (XLRS) (*35*), and the severity correlated with viral load. For eight of the nine patients with XLRS, no efficacy was observed, likely due to dilution of the vector as it diffuses throughout the vitreous cavity and anterior chamber. Dilution decreases transduction potential to target cells (e.g., RPE) (*42*). Furthermore, free diffusion throughout the eye increases likelihood of AAV entry into the systemic circulation. Others have addressed this problem by generating novel AAV serotypes through directed evolution (*43*). The 4D-R100 AAV, for example, was invented to traverse the ILM following intravitreal injection at lower doses than is required of other serotypes. It is currently in clinical trials to treat choroideremia (NCT04483440), XLRP (NCT04517149), and neovascular AMD (NCT06864988 and NCT05197270). Although it is our opinion that neither suprachoroidal nor intravitreal routes are promising alternatives to the subretinal route, it would be interesting to compare our epiretinal approach to conventional routes using the 4D-R100.

One concern of our epiretinal fibrin approach is that this pilot study was performed only in pigs. However, our team has treated one patient with a large chronic, recurrent macular hole by placing a 5-mm fibrin hydrogel on the epiretinal surface overlying the macular hole (fig. S3). The hydrogel was constructed the same day as the surgery using an Evicel tissue glue kit, a sterilized mold, and a sterilized 5-mm punch. Aprotinin and trypan blue were not used during this process. The gel was easy to insert, lay flat on the retina surface, and remained in place postoperatively. This gel, like the gels in our pigs, degraded completely within days and caused no clinically notable inflammation. This surgery resulted in improved anatomy with a smaller macular hole in a more favorable configuration. The patient has remained stable for over 26 months. This single success in a human patient using a similar fibrin hydrogel demonstrates the potential for this approach in humans.

The present study reports animal data showing successful placement in vivo of hydrogel encapsulated AAV and successful transduction of the target tissues and cells. The hydrogel used is produced from fibrin and degrades rapidly when placed on the epiretinal surface. Although the present study has focused on ophthalmic indications and was driven by the adverse events observed in humans receiving both approved and experimental gene therapies for eye disease, the technology is portable. The ability to slow viral release and confine the vector dose to a specific location in a tissue could be applied to other organ systems. The slow release in the eye appears to potentially reduce the needed titer, limit diffusion of the therapy to adjacent tissues through the vasculature, and reduce the frequency of inflammation. Although the retina is often considered an immune privileged site, the vitreous cavity is not. Given the favorable outcomes of epiretinal fibrin gene therapy delivery, we envision future studies examining AAV-containing fibrin hydrogels applied to various nonophthalmic tissue compartments, including the subcutaneous, joint cavity, perivascular, pericardial, and subarachnoid spaces. Testing the efficacy of this hydrogel AAV delivery platform compared to conventional methods in disease animal models will advance this innovation closer to clinical translation.

## MATERIALS AND METHODS

### Study design

The objective of this research was to develop a safer and more effective method of delivering gene therapy to the RPE both centrally and peripherally without subretinal injection. Our prior study in pigs showed safety of fibrin gels in the eye (*24*). Therefore, using 11 female domestic pigs that were 25 to 30 kg in weight, we performed a proof-of-concept procedure using fibrin hydrogels containing AAV2-*GFP*. The characterization of these gels and the outcomes of this pilot pig study are included. To compare this fibrin hydrogel approach to the conventional subretinal and intravitreal approaches, five additional female domestic pigs of similar size underwent vitrectomy with subretinal administration of AAV2-*GFP* solution without hydrogel, and three additional female domestic pigs of similar size underwent intravitreal injection of AAV2-*GFP* solution (table S1). All experimental animals received similar viral titers.

### AAV2-*GFP* vectors

Recombinant AAV2 vectors expressing enhanced GFP under control of the cytomegalovirus (CMV) promoter were generated at the University of Iowa as previously described (*44*, *45*). Recombinant AAV2/Quad vectors expressing GFP under control of the CMV promoter generated at the University of Florida Ocular Gene Therapy Core were also used. The vector and titer used for each pig are included in table S1.

### Production of fibrin-AAV hydrogels

All steps in production of fibrin hydrogels were performed aseptically. Fibrin hydrogels were produced using 4-ml Tisseel tissue glue kits (Baxter NDC#00338-4302-04). The fibrinogen was resuspended in sodium citrate prepared from a clinical anticoagulant solution (Fenwal/NDC-0942-9504-10) diluted to 0.01 M with sterile water (Gibco) for injection rather than the included resuspension buffer, which contains aprotinin and was omitted to promote rapid degradation of the gel in vivo. Thrombin was reconstituted with the thrombin solution from the same Tisseel Kit, and the vials incubated in a 37°C water bath overnight. The following day, 0.6 ml of sterile tissue culture grade 0.4% trypan blue (Thermo Fisher Scientific, 15250061) was added to 2 ml of the resuspended fibrinogen and thrombin solutions to slow the polymerization reaction (*26*). The virus solution (4 × 10^11^ vg) was added to the thrombin solution for fibrin gels containing AAV. The fibrinogen solution was then drawn into an 11-ml syringe and the thrombin solution into a 1-ml syringe. The syringes were placed in an 11:1 ratio FibriJet Ratio Applicator Assembly. The gelation solution was then dispensed through a FibriJet Blending Connector with Mixer (Nordson Medical) and 18-gauge cannula into a custom mold (Meddux, CO). The molds were then incubated at 37°C for 3 hours to cure. Subsequently, molds were opened and top plates containing the polymerized gels (Fig. 2A and fig. S1) were transferred to a 4-well culture dish (Thermo Fisher Scientific) in sterile phosphate-buffered saline (PBS). The resulting gel is 15.25 mm by 58.42 mm by 0.2 mm with a final concentration of 30 mg/ml of fibrin. Within 1 hour of curing, the gel blank was cut to various sizes/shapes using punches. Oval-shaped gels measuring 1.5 mm by 5.1 mm by 0.2 mm were punched for in vitro characterization (e.g., degradation assay and immunostaining) and surgical implantation in the pig, whereas 3-mm circular punches of 0.2 mm in thickness were used for the in vitro assay and cell culture experiments. Exact AAV2 titers per gel punch varied for each experiment, and specific titers are included in table S1.

### Fibrin gel degradation protein assay

Oval-shaped gels of 1.5 mm by 5.1 mm by 0.20 mm were degraded at room temperature in 50 μl of sterile water containing 0.14 μg of plasminogen (specific activity of plasmin > 12,500 pM/min per microgram, R&D Systems) activated with 0.19 μg of tissue plasminogen activator (tPA; 300 U/μg, Sigma-Aldrich) for time points between 0 and 48 hours. At the end point, any remaining gel was removed from the solution and photographed. The solution was stored at −20°C before assay for total protein using a BCA protein assay kit (Abcam) according to the manufacturer’s instructions and adjusted to account for the plasmin and tPA in the solution and normalized against total protein released from gels degraded to 100%.

### AAV2 ELISA for assessment of viral diffusion

Fibrin gels containing 1 × 10^9^ vg AAV2-*GFP* and measuring 3 mm in diameter were placed into individual wells of a 96-well plate with 100 μl of ELISA buffer per well. Gels and supernatant (i.e., containing any AAV eluted from gels) were harvested from the wells in triplicate at multiple time points (1, 24, and 48 hours) and then frozen and stored at −80°C. Once all time points were collected, the gels were degraded in 25 μl of sterile H_2_O containing 2.3 μg of plasminogen (>12,500 pM/min per microgram, R&D Systems) activated with 0.19 μg of tPA (300 U/μg, Sigma-Aldrich) at 37°C for 3 hours. Gel solutions were diluted to a total volume of 100 μl. Using the Progen AAV2 Xpress ELISA kit (Progen Biotechnik), the gel solutions (containing any retained AAV in gels) and supernatants (containing eluted AAV) were analyzed for viral capsids present.

### Cell culture

ARPE-19 cells were maintained at 37°C in a 95% air/5% CO_2_ incubator in Dulbecco’s modified Eagle’s medium (DMEM)/F12 containing 10% fetal bovine serum (FBS) and 1% antibiotic/antimycotic. AAV-encapsulated fibrin gels were prepared as described above and punched to 3 mm in diameter. Each gel contained 1 × 10^9^ vg AAV2-*GFP* per punch. These punches were directly added to individual wells of a confluent 96-well plate of ARPE-19 cells. Media were changed 2 days after addition of the hydrogels or viral solution. The plate was monitored daily for GFP expression for 1 week. Each well of the plate was imaged using a Nikon Ti inverted fluorescence microscope and charge-coupled device (CCD) camera using Nikon Elements software.

### Flow cytometry

To determine the total GFP-expressing ARPE-19 cells at 72 hours and 1 week after hydrogel placement, flow cytometry was performed at the Mayo Clinic Microscopy and Cell Analysis Core Facility. To isolate the ARPE-19 cells, TrypLE (Gibco) solution was used to release the cells. ARPE-19 cells from a 3-well plate were pooled and placed into 96-well U-bottom plates with DMEM/F12. The plate was then washed twice with calcium/magnesium-free (CMF)–PBS and stained with Ghost Red dye for cell viability. The cells were washed two additional times with CMF-BPS and stored in fluorescence-activated cell sorting (FACS) buffer (5% FBS and 0.1% sodium azide in CMF-PBS) in the dark until flow cytometry was performed. Flow cytometry was performed using a ZE5 flow cytometer (Bio-Rad) using SSC (488/10), Ghost Red (775/50), and an enhanced GFP (525/35) laser line. For the 72-hour time point, three different runs were performed and averaged (nine wells represented). For the 1-week time point, nine different runs were performed and averaged (27 wells represented).

### Gel thickness measurement

Immediately after gel preparation, the metal backing and silicon gasket were removed (fig. S1). A 14 × 5 grid was drawn along the plastic slide to mark areas for OCT measurement (Fig. 2). Columns were spaced 5 mm apart, and rows were spaced 4 mm apart. During imaging, the plastic slide holding the fibrin gel was placed in a clear 10-cm petri dish with sterile PBS. Gel thickness was measured at the intersections of the grid using an OCT imaging system (Lumedica OQ Labscope; version 2.0). Uniformity of thickness was determined from B scans of the gels at all indicated locations.

### IF staining of fibrin hydrogels

Oval-shaped fibrin hydrogels frozen in optimal cutting temperature compound were sectioned to 10 μm and frozen for storage; sections were held at room temperature overnight for dehydration before staining. Slides were stained with rabbit anti-AAV polyclonal antibody (Invitrogen) and a mouse monoclonal anti-Fibrin antibody 59D8 (Millipore) for 1 hour at room temperature. Slides were washed and reacted with goat anti-rabbit conjugated to Alexa Fluor 488 (Invitrogen) and goat anti-mouse conjugated to Alexa Fluor 568 (Abcam) for 1 hour at room temperature. Slides were washed and mounted with Fluoromount (Invitrogen). For controls, secondary antibodies were used without primary antibodies.

### Electron microscopy

Both SEM and TEM were performed at the Mayo Clinic Microscopy and Cell Analysis Core as previously described (*23*, *26*).

### Mechanical testing

Mechanical testing was conducted using a spherical indenter (radius = 0.25 mm) on the MicroTester G2 (CellScale, Waterloo, ON) to determine the Young’s modulus at the Mayo Clinic Biomechanics Core Facility. Each gel specimen was mounted on a microscope slide and submerged in a bath of heated PBS (37° ± 1°C) during the testing. Gels were indented up to 10% of thickness with the displacement and resulting loading force data used to fit the Hertz contact model

$$F=\frac{4}{3}\frac{E}{1-v^{2}}R^{0.5}d^{1.5}$$

where *F* is the normal force being applied to the gel, *d* is the displacement, *R* is the radius of the indenter, and *v* is the material’s Poisson’s ratio (approximated to be 0.5). *E* is the Young’s modulus, which was solved for in MATLAB.

### Animals

All animal procedures were approved by the Institutional Animal Care and Use Committee (IACUC; protocol nos. A00007909-00 and A00006657-23) of Mayo Clinic and conducted in accordance with the Association for Research in Vision and Ophthalmology Statement for the Use of Animals in Ophthalmic and Vision Research. Nineteen 2- to 3-month-old female domestic pigs (*Sus scrofa domesticus*) weighing 20.4 to 35.8 kg were used for this study. For all animals, the right eyes were designated as the experimental/operative eyes, whereas the left eyes (fellow eyes) were used only as control eyes for postmortem imaging and histological comparison.

### Vitrectomy with epiretinal fibrin-AAV gel placement

Surgeries were performed as before (*24*) with the following modifications. A 1- to 5-mm lateral canthotomy was performed on the right eye to improve exposure, and a 5-ml retrobulbar block of cefazolin (100 mg/ml) was administered into the subtenon’s space superotemporally to proptose and stabilize the eye.

Three-port triamcinolone-assisted 25-gauge pars plana vitrectomy using a Constellation Vitrectomy System (Alcon, Fort Worth, TX) and wide-angled, noncontact Biom fundus lens (Oculus Surgical, Wetzlar, Germany) was performed by B.A.S., a vitreoretinal surgeon, with careful separation of the posterior hyaloid in the area centralis. Eye pressure was maintained using balanced salt solution (BSS) infusion. We intentionally did not perform a peripheral vitrectomy on any pig as to provide vitreous support to the peripheral retina.

For eyes receiving an epiretinal implant (*n* = 11), 1 to 2 ml of PFO was injected over the central retina. A 4-mm sclerotomy was then made with an MVR blade, and an argon laser on the Constellation Vitrectomy System was applied to the choroid before entering the globe with a keratome blade. The oval-shaped fibrin gel containing ~2 × 10^9^ vg AAV2-*GFP* was inserted using a custom instrument (*24*, *46*) through the sclerotomy and beneath the PFO bubble. The gel was noted in every case to lie flat on the epiretinal surface. Autologous pig serum (100 to 200 μl), which was obtained and processed same day as the surgery, was injected around the gel while the PFO was still present. This was allowed to set for 10 min before removing the PFO by fluid-air exchange. The gel remained in place for all 11 epiretinal surgeries. Nonexpansile (20%) SF6 gas was then injected. The sclerotomies were sutured with 8-0 Vicryl sutures, when indicated, to ensure airtight closure of all sclerotomy sites. The canthus was sutured with 4-0 chromic gut interrupted sutured. Gentamicin (0.3%) drops were topically administered to the right eye three times daily for 5 days postoperatively.

### Vitrectomy with subretinal viral solution administration

For eyes receiving subretinal AAV2-*GFP* solution (*n* = 5), vitrectomy was performed as described for the epiretinal group, but no gel was inserted. A 38-gauge polyimide subretinal cannula (38g PolyTip, MedOne, Sarasota, FL) was used to create a subretinal bleb in the area centralis with the AAV solution (2 × 10^9^ vg in 300 μl) with slow manual injection using a skilled assistant. The eye pressure was lowered to 10 mmHg during subretinal bleb formation. A fluid-air exchange was performed before closure as previously described. There was no PFO injection or 4-mm sclerotomy made for the subretinal group. No laser or steroid was applied for any of the experimental eyes.

### Viral solution administration via intravitreal injection

For eyes receiving intravitreal AAV2-*GFP* solution (*n* = 3), a 0.5-inch-long 30-gauge needle (BD Biosciences) was inserted through the pars plana, 2.75 mm posterior to the limbus, in the superotemporal or inferotemporal quadrant of the right eye. Each pig received a single 50-μl injection with 2.5 × 10^9^ to 2.75 × 10^9^ vg.

### Color fundus photography, OCT, and OCT-A

The pig was anesthetized using isoflurane as detailed in Gandhi *et al.* (*24*) for each postoperative examination. Eye drops were instilled for dilation and topical anesthesia as above. Color fundus photos, OCT, and OCT-A were performed at 5 to 7 days, 2 weeks, and 1 month postoperatively. Fundus photographs were obtained using a custom-made video indirect ophthalmoscope. Images were processed using Photoshop (Adobe, San Jose, CA). OCT, OCT-A, and infrared SLO were performed using the Optovue Avanti OCT Angiovue System (Visionix; North Lombard, IL).

### Postoperative evaluation of infection

For one intravitreal pig (table S1), an anterior chamber tap was performed at the beginning of postoperative week 2 given severe vitritis and anterior chamber inflammation was present. This was performed with a 30-gauge needle at the corneal limbus while the animal was under general anesthesia. This sample was sent to IDEXX Laboratories Inc. (Westbrook, ME) for Gram staining and bacterial and fungal cultures.

### Postmortem exam and histology

Pigs were euthanized by rapid intravenous injection of a pentobarbital solution [FATAL-PLUS, Vortech; 1 ml/per 10 pounds (4.54 kg) of body weight] at 1 month postoperatively. Eyes were enucleated and immersed either in PBS for immediate imaging or in Davidson’s fixative for later processing.

For the eyes immersed in PBS (*n* = 6 epiretinal and *n* = 1 subretinal), the eye was immediately dissected within 1 hour of enucleation. The cornea, lens, portion of the remaining vitreous, and optic nerve were collected and stored at −80°C. These eyes were cut to allow flat mounting for examination using a Leica M165FC fluorescence stereomicroscope. Mounted posterior segments (i.e., retina and sclera) were cut into regions of interest and immediately placed in optimal cutting temperature compound and frozen at −80°C. Cryosections were cut to 8 μm. Slides were mounted with a 4′,6-diamidino-2-phenylindole (DAPI)–containing mounting medium (Vector Laboratories) before imaging.

For H&E, IHC, and IF staining, pig eyes fixed in Davidson’s fixative were processed into paraffin (*n* = 4 epiretinal, *n* = 3 subretinal, and *n* = 3 intravitreal) and sectioned at 5 μm as described previously (*24*). Sections stained with H&E were photographed using a Nikon E-600 microscope with a color CCD camera and NIS-Elements Software (Nikon) or scanned using an Aperio AT2 microscope slide scanner.

IHC staining was performed as described previously (*47*, *48*) with the following modifications: Following deparaffinization, heat-mediated antigen retrieval was performed using an Aptum Biologics 2100 Antigen Retriever in with R-buffer B (Leica). IHC staining was performed using Goat anti-GFP (Abcam), Rabbit anti-IBA1 (Invitrogen), or Rabbit anti-GFAP (Novus) with the VectaStain Elite ABC-HRP and Vector VIP kits (Vector Labs) according to the manufacturer’s instructions. Nuclei were counterstained with methyl green (Vector Laboratories).

For IF staining, deparaffinized sections were subject to heat-mediated antigen retrieval as above and stained with Goat anti-GFP (Abcam), Rabbit anti-GFAP (Novus), and Rabbit anti-IBA1 (Invitrogen). Secondary antibodies, applied in sequence to avoid cross-reactivity, were Donkey anti-goat conjugated to Alexa Fluor 488 (Abcam), followed by Goat anti-rabbit conjugated to Alexa Fluor 568 (Abcam). Tissue autofluorescence was mitigated using the Vector TrueView Autofluorescence Quenching kit (SP-8400) according to the manufacturer’s instructions. Nuclei were then stained with DAPI (1:1000).

### Real-time qPCR for detection of AAV in postmortem samples

After eye enucleation, samples of the cornea, lens, vitreous, and optic nerve were collected. A sample of the liver was also harvested. Genomic DNA was extracted from these tissues using the Quick-DNA Microprep Plus Kit (Zymo Research) and stored at −80°C until use.

qPCR assays were performed using a QuantStudio 5 Real-Time PCR Instrument (Applied Biosystems). The amplifications were carried out in 20-μl reaction solutions containing 10 μl of 2x Taqman Universal PCR Mastermix (Thermo Fisher Scientific, 4304437) with 1 μl of Taqman Gene Expression eGFP Probe (250 nM; Thermo Fisher Scientific, assay ID: Mr04329676_mr), each primer (900 nM; Thermo Fisher Scientific, 4331182), and 10 ng of genomic DNA in 9 μl of sterile water. The PCR thermal cycling conditions were as follows: 50°C, 2 min; 95°C, 10 min; 95°C, 15 s; and 60°C, 1 min for 40 cycles. Each assay was performed in quadruplicate to check for reproducibility along with a GFP-positive control and no template–added control. Data were analyzed using QuantStudio Software v1.5.1 and compared to titrated AAV spike-ins (fig. S2) to establish a limit of detection as before (*49*).

### Human macular hole repair using fibrin hydrogels

A 62-year-old female presented with a chronic macular hole in the left eye. Fully informed consent was obtained to perform macular hole repair, explaining the risks, benefits, and alternatives to pars plana vitrectomy with membrane peeling, peeling of the internal limiting lamina, and fibrin gel patching of the macular hole. A 23-gauge pars plana vitrectomy was performed by R.I., and the vitreous base was shaved 360°. The ILM was removed using intraocular forceps after staining with diluted indocyanine green. PFO was then injected overlying the macula. A 5-mm sclerotomy was created superotemporally. A 5-mm-diameter fibrin gel was inserted into the eye using 23-gauge intraocular forceps and placed onto the epiretinal surface under the PFO overlying the macular hole (fig. S3). A direct PFO–to–silicone oil (1000 centistokes) exchange was performed, and the sclerotomies were sutured closed. Serial OCT images and color fundus photography were obtained in clinic starting at postoperative day 1 to assess the gel degradation and the macular hole size and configuration over time. Patient consent and Mayo Clinic Institutional Review Board approval were obtained (protocol #25-003256) to retrospectively report the outcomes of her care.

### Statistical analyses

Statistical analyses were conducted using a two-sample two-tailed Mann-Whitney *U* test to compare two cohorts, including the Young’s modulus between fibrin gels with and without aprotinin and the percentage of GFP-positive ARPE-19 cells based on flow cytometry at 72 hours and 1 week. The Kruskal-Wallis one-way analysis of variance (ANOVA) test with Dunn’s post hoc analysis was performed to compare the average gel thickness across three different gels. All statistical tests were performed using GraphPad Prism 4.0b for Macintosh (GraphPad Software, San Diego, CA). All values of gel measurements (e.g., thickness) were reported as averages ± SEM. The fibrin gel protein degradation curves were fit to a second-order polynomial (quadratic) formula and compared using least-squares regression method. GraphPad Prism 4.0b for Macintosh, Adobe Photoshop, and Microsoft Excel were used to generate figures. Significance for comparisons was defined as *P* < 0.05 for all analyses.
