# Supplementary material for: Retinal gene therapy using epiretinal AAV-containing fibrin hydrogel implants
Source: Sci Adv. 2025 Sep 5;11(36):eadv7922. doi: 10.1126/sciadv.adv7922 (PMC12412667; doi:10.1126/sciadv.adv7922)
Supplement: Supplementary file 1 — Figs. S1 to S3 Tables S1 and S2 Legend for movie S1 [file sciadv.adv7922_sm.pdf]

Science Advances
AAAS

Supplementary Materials for

Retinal gene therapy using epiretinal AAV-containing fibrin hydrogel implants

Brittni A. Scruggs *et al.*

Corresponding author: Alan D. Marmorstein, marmorstein.alan@mayo.edu

Sci. Adv. **11**, eadv7922 (2025)
DOI: 10.1126/sciadv.adv7922

The PDF file includes:

Figs. S1 to S3
Tables S1 and S2
Legend for movie S1

Other Supplementary Material for this manuscript includes the following:

Movie S1

Supplementary Material

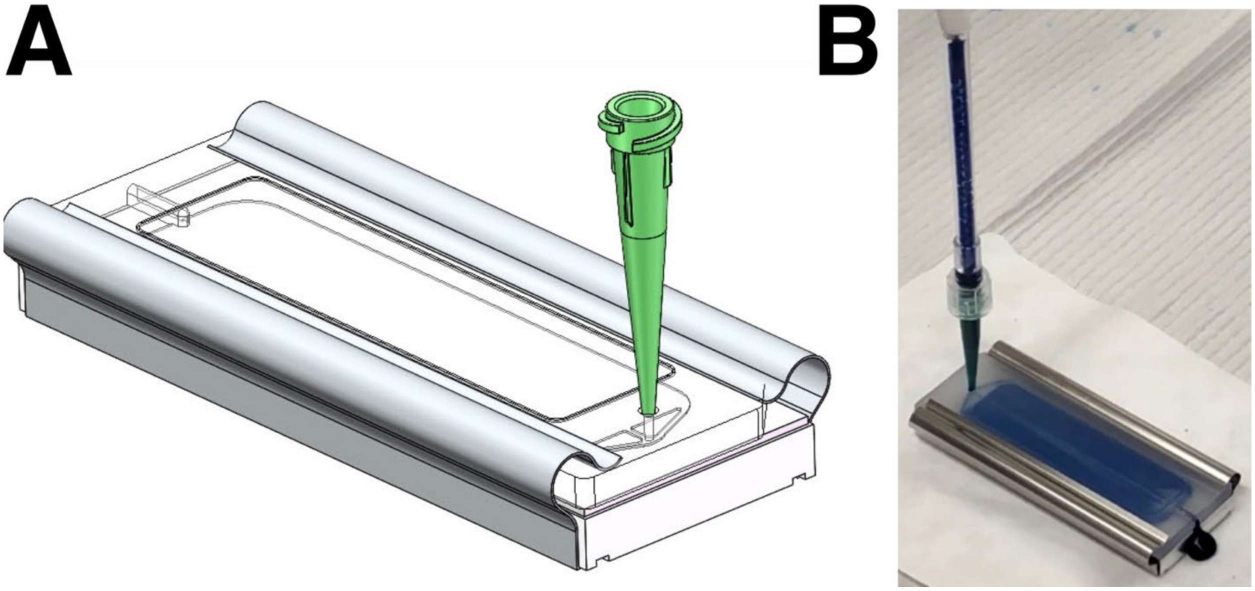

Supplementary Fig. 1. **Fibrin hydrogel construction.** (**A**) Drawing of a fully assembled fibrin casting mold with green 18-gauge cannula tip placed over injection port hole. (**B**) Image of injected AAV2/fibrinogen solution into the casting mold to form a complete fibrin gel blank with excess gelation solution observed coming from the pressure release port.

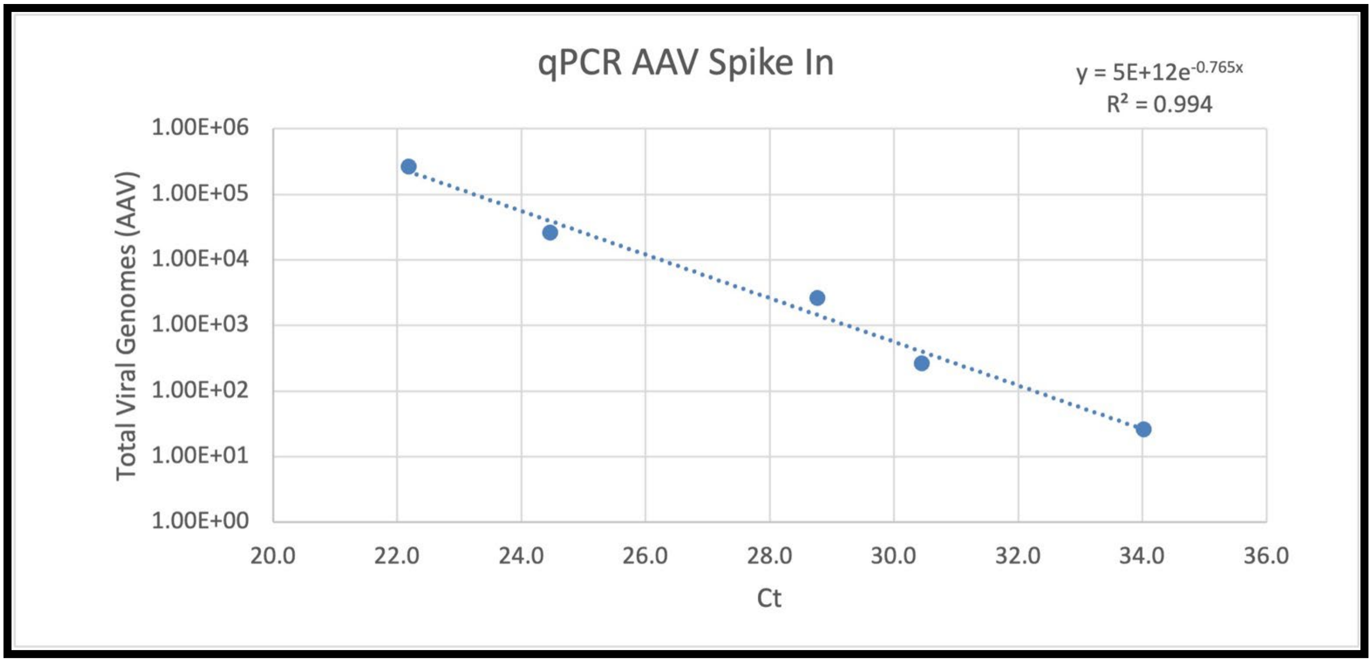

Supplementary Fig. 2. qPCR AAV Spike In Data. To determine the limit of detection of AAV, AAV2/2-*GFP* was added to 10 ng of DNA isolated from the liver of a naïve pig in serial 10-fold dilutions from 2.7 x 10^6 vg to 2.7 x 10^0 vg. Ct diminished linearly as a function of vg concentration over the range of 2.7 x 10^6 to 2.7 x 10^1 vg with R^2 = 0.994. Spike in with <2.7 x 10^1 vg did not consistently rise above threshold through 40 cycles. From these data we conclude that the limit of detection of this assay is 27 vg DNA.

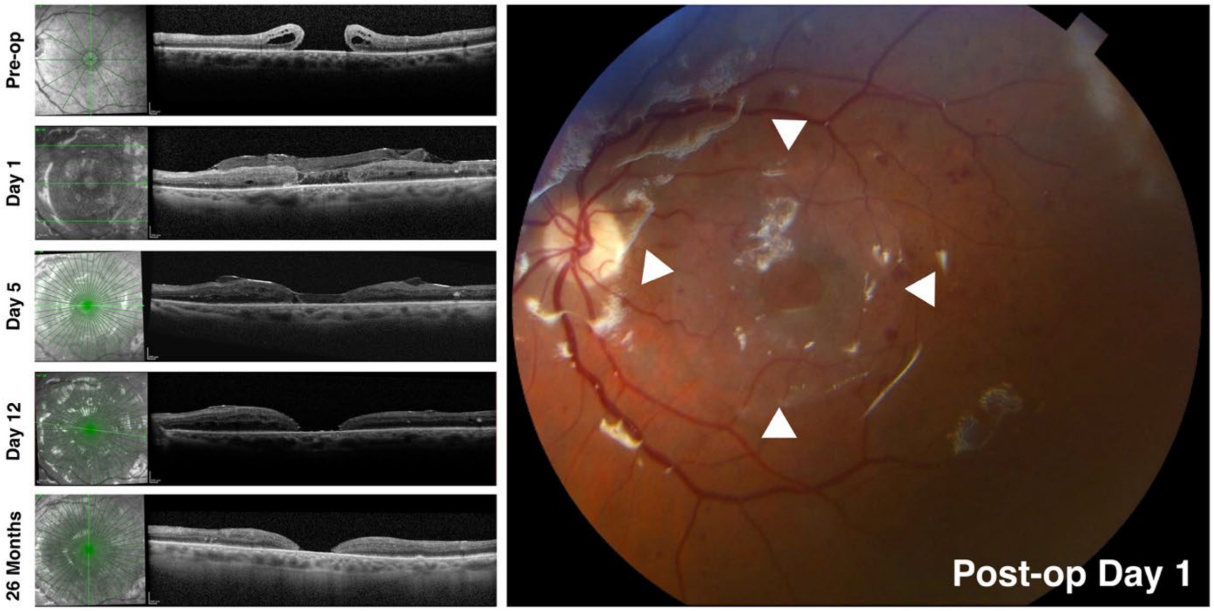

Supplementary Fig. 3. **Human macular hole surgery with fibrin hydrogel.** A 5 mm diameter fibrin gel (right, arrows) was placed on the epiretinal surface overlying a chronic, recurrent macular hole, refractory to conventional surgical repair methods. Serial OCT images (left) show the large macular hole pre-operatively (top left) and the gel on the retinal surface at day 1 post-operatively. The gel appeared mostly degraded by day 5 post-operatively and was completely degraded by day 12. There was no clinical inflammation peri-operatively, and there were no short-term or long-term complications. Noted to be smaller after surgery, the hole had an improved configuration without intraretinal fluid and without elevated retinal edges up to 26 months post-operatively.

Pig Number		Gene Therapy			Sample Processing		Transduction Efficiency		Outcomes and Adverse Events		
		Viral Vector	Route	Concentration (vg)	Cryo-section	Paraffin Section	Local/ Central	Peripheral	CRA	Vitreous Opacities	Surgical Complications
1	171	AAV2/ Quad	Epiretinal	$1.56x10^9$	+	-	+	+	-	-	Abscess localized to the sclerotomy site, treated with antibiotics
2	262	AAV2/ Quad	Epiretinal	$1.15x10^{10}$	+	-	+	+	-	+	None
3	263	AAV2/ Quad	Epiretinal	$1.15x10^{10}$	+	-	+	+	-	+	None
4	264	AAV2/ Quad	Epiretinal	$1.15x10^{10}$	+	-	+	+	-	+	Small peripheral retinal detachment noted at time of euthanasia, not near surgical site
5	315	AAV2	Epiretinal	$1.60x10^9$	+	-	+	+	-	+	None
6	323	AAV2	Epiretinal	$1.60x10^9$	+	-	+	+	-	-	None
7	384	AAV2	Epiretinal	$2.50x10^9$	-	+	+	+	-	-	None
8	385	AAV2	Epiretinal	$2.50x10^9$	-	+	+	+	-	-	None
9	491	AAV2	Epiretinal	$7.00x10^9$	-	-	+	+	-	+	None
10	492	AAV2	Epiretinal	$7.00x10^9$	-	+	+	+	-	-	Gas cataract developed following surgery
11	495	AAV2	Epiretinal	$7.00x10^9$	-	+	+	+	-	-	None
12	170	AAV2/ Quad	Subretinal	$2.25x10^9$	+	-	+	-	+++	+	Severe atrophy throughout bleb
13	383	AAV2	Subretinal	$2.50x10^9$	-	+	+	-	-	+	Small peripheral corneal abrasion with resultant stromal opacity/scar
14	424	AAV2	Subretinal	$2.50x10^9$	-	+	+	-	+++	++	Severe atrophy throughout bleb, mild clinical inflammation
15	425	AAV2	Subretinal	$2.50x10^9$	-	+	+	-	-	++	Mild clinical inflammation
16	706	AAV2	Subretinal	$2.66x10^9$	-	-	+	-	-	-	None
17	428	AAV2	Intravitreal	$2.50x10^9$	-	+	+	+	-	++	Cataract developed at month 1. Clinical inflammation present with mild to moderate vitritis at week 1. No anterior chamber inflammation. Resolved with topical steroids.
18	429	AAV2	Intravitreal	$2.50x10^9$	-	+	-	-	-	+++	Cataract developed at month 1. Clinical inflammation present with moderate to severe vitritis and anterior chamber inflammation at week 1. Resolved with topical and systemic steroids. IDEXX testing performed on aqueous humor, negative for infection.

19	514	AAV2	Intravitreal	$2.75x10^9$	-	+	+	+	-	-	Mild retinal vasculitis (vascular sheathing) but no clinical signs of anterior chamber inflammation or vitritis

Supplementary Table 1. Overview of animal surgeries and outcomes. For all pigs included in this study, details of gene therapy vector, surgical route, AAV2 titer administered, post-mortem eye processing, transduction sites, and adverse events are provided. (+) indicates Yes or present but without clinical significance. (++) indicates clinically significant but treatable. (+++) indicates severe clinical implications.

[a] For pigs 491 and 706, eyes were used for whole mount brightfield and fluorescence imaging only. These eyes were not processed for histology purposes.

[b] The ability to transduce RPE cells locally at the site of implant or bleb placement (*i.e.,* centrally) and in the periphery (*i.e.,* anterior to the equator) was determined by IHC, GFP fluorescence of unfixed, cryosections (without GFP antibody), IF, and/or FITC imaging, depending on each sample's processing method.

[c] For eyes with vitreous opacities noted as (+) with "no clinical significance," these vitreous cells were only noted on OCT imaging and *not* on clinical exam. No treatment was needed.

		Right Eye					Left Eye						
**Pig**	**Surgery**	**Iris**	**Lens**	**Cornea**	**Vitreous**	**Optic Nerve**	**Iris**	**Lens**	**Cornea**	**Vitreous**	**Optic Nerve**	**Liver**	**NTC**
171	Epi-Imp	–	–	–	NA	NA	–	–	–	NA	NA	NA	Clean
262	Epi-Imp	–	–	–	–	–	–	–	–	–	–	–	Clean
264	Epi-Imp	–	–	–	–	–	–	–	–	–	–	NA	Clean
315	Epi-Imp	NA	–	–	<30000	–	–	–	NA	–	–	–	Clean
323	Epi-Imp	–	–	–	<30000	–	–	–	–	–	–	–	Clean
384	Epi-Imp	–	–	–	–	–	NA	NA	NA	NA	NA	NA	Clean
385	Epi-Imp	–	–	–	–	–	–	–	–	–	–	–	Clean
492	Epi-Imp	–	–	–	<300	–	–	–	–	–	–	NA	Clean
495	Epi-Imp	NA	NA	NA	NA	NA	NA	NA	NA	NA	NA	–	Clean

Supplementary Table 2. AAV detection after epiretinal fibrin gene therapy in post-mortem pig samples. Viral (AAV2) detection by qPCR in post-mortem right eye (surgical eye), left eye (fellow eye), and liver samples in pigs that received epiretinal implants with AAV2 virus (Epi-Imp). Not Detected: – ; Not available: NA; No template control: NTC. Limit of Detection was 27 viral genomes, which correlates to Ct = 34.

Supplementary Video 1. Intraoperative video of the epiretinal placement of an AAV-containing fibrin hydrogel in a pig.
